# Comparative Analysis of Blood MMP-9 Concentration in Alcohol- and Opioid-Addicted Patients

**DOI:** 10.3390/diseases13020030

**Published:** 2025-01-24

**Authors:** Tamar Kartvelishvili, Nelly Sapojnikova, Nino Asatiani, Lali Asanishvili, Victor Sokhadze, Nestan Sichinava, Zaza Chikovani

**Affiliations:** 1 Andronikashvili Institute of Physics, I. Javakhishvili Tbilisi State University, Tbilisi 0162, Georgia; 2 Narcological Clinic “Nishati”, Tbilisi 0186, Georgiazazachik@gmail.com (Z.C.)

**Keywords:** MMP-9, alcohol addiction, opioid addiction, plasma

## Abstract

Background/Objectives: In brain physiology and disease, MMP-9 is a significant and apparently peculiar factor. Numerous studies have implicated neuroinflammatory processes involving MMP-9 in the pathophysiology of addiction. This study aims to evaluate plasma MMP-9 level as a biomarker for the stages of alcohol and opioid addiction. Methods: The case subjects were patients with opioid and alcohol addiction. The quantitative assessment of MMP-9 plasma concentration was performed using monoclonal antibodies against human MMP-9. Results: MMP-9 levels in the plasma of patients with alcohol and opioid dependence differ from MMP-9 concentrations in apparently healthy donors. During the intoxication stage, MMP-9 concentrations in individuals with alcohol and opioid dependence are similar and higher than in the control group. While the MMP-9 level is close to the control level after alcohol withdrawal, it stays increased during opioid withdrawal. When MMP-9 levels in plasma were measured in three distinct intoxicated states (light, moderate, and heavy) in cases of alcohol addiction, the results were all similar. Two distinct opioid intoxicated states (methadone and buprenorphine) and three withdrawals—following methadone, buprenorphine, and heroin abuse—were associated with high MMP-9 levels.

## 1. Introduction

Metalloproteinases, in particular matrix metalloproteinases (MMPs), are essential for many physiological and pathological processes in organisms [1,2]. MMPs, proteoglycans, glycosaminoglycans, collagen and elastin, fibronectin and laminin, and structural and adhesion proteins represent a number of the components that compose the extracellular matrix (ECM) [3]. For cell migration, the ECM serves as a movement track, an anchoring site, or a physical barrier. MMPs, the ECM-remodeling peptidases, can degrade almost all components of the extracellular matrix. The critical role of extracellular MMPs is in modulating cell-to-cell and cell-ECM contacts, which regulate vital tissue homeostasis [4].

MMPs are produced by endothelial cells, microglia, oligodendrocytes, neurons, and astrocytes in the central nervous system (CNS) [5]. They regulate the signaling cascade during synaptic dysfunction, disruption of the blood–brain barrier (BBB), neuroinflammation, or neuronal death [6]. MMP activation is strictly regulated, and disruption of this regulation causes specific pathologies in neurodegenerative disorders (NDs) [7]. Transcription, post-transcription, translation, post-translation, and epigenetic regulations; proenzyme activation through deletion of the proenzyme domain; interaction with tissue inhibitors of metalloproteinases (TIMPs); pericellular and intracellular compartmentalization; and oxidative stress are factors that control MMP expression and activity [8,9,10,11,12].

MMPs are classified by domain structure into eight groups, five of which are secreted and three are membrane-associated MT-MMPs. The secreted matrix metalloproteinase-9 (MMP-9) is one of the most complex MMPs [13]. MMP-9 is a 92 kDa type IV collagenase that is a member of the calcium-dependent and zinc-containing endopeptidase family. It is also known as gelatinase B because of its ability to cleave gelatin. The biochemistry and molecular biology of MMP-9 have been reviewed in detail by Vandooren et al. [14]. MMP-9 normally functions in the extracellular region, where it is secreted as an inactive proform, or zymogen, with a propeptide of about 10 kDa protecting the enzymatic active site [15]. The activation of MMP-9 zymogen, which involves the removal of the propeptide, is dependent on a conformational shift that can be triggered by different mechanisms [16]. Given the restricted and time-dependent activity of MMP-9, the tight regulation of its expression appears to be indispensable in terms of sustaining physiological conditions [17].

The neuroinflammatory response and the interaction between peripheral inflammation and neuroinflammation have been shown to be regulated by MMP-9. MMP-9 has been identified as a key inflammatory mediator [18], is released by neurons and activated immune cells [19,20], and interacts with chemokines and cytokines [21]. Blood–brain barrier disturbances, such as the breakdown of tight junction proteins and the capillary basement membrane, are linked to MMP-9 overexpression [22]. Increased oxidative stress and neuroinflammation, as well as decreased interneuron creation, were caused by MMP-9 overexpression [23].

Due to their ability to induce oxidative stress, alcohol and opioids can change the way that membrane regulatory proteins in various neurotransmitter systems send nerve impulses [24]. Alcohol is a highly addictive chemical. Consuming alcohol is well recognized as an avoidable contributory factor to the development of several diseases, affecting the liver, heart, brain, and gastrointestinal tract. It also has the potential to exacerbate some NDs linked to aging-related changes [25]. The overall number of overdose deaths and opioid use disorders (OUDs) has increased to levels that were not previously observed.

Numerous lines of evidence connect microglial activation and pro-inflammatory cytokine signaling to opioid craving and reward processing vulnerability [26,27]. The ECM signaling proteins, MMPs, are implicated in opioid reward and addiction [28,29]. MMP-9 has been connected to a number of addiction disorders. Chronic opium and methamphetamine addiction alters MMP-9 activity and concentration levels in serum [30]. Following opioid administration, increases in MMP-9 expression may be essential for both drug-induced neuronal plasticity and behavioral tolerance and dependence [31]. MMP-9 activity in the hippocampus was reduced by acute ethanol intoxication, which also limited the development of spatial memory [32]. Moreover, chronic alcohol abusers were shown to have elevated serum MMP-9 concentrations [33].

The evaluation of the plasma MMP-9 level as a biomarker of the stages of alcohol and opioid addiction is the goal of the present study. MMP-9 levels in the blood are assessed throughout the intoxication and withdrawal stages of alcohol and opioid addiction. Blood MMP-9 concentration as a non-invasive instrument could be beneficial for tracking disease risk factors due to body fluids exhibiting noticeable cellular alterations.

## 2. Materials and Methods

### 2.1. Clinical Study

The case subjects were patients with opioid and alcohol addiction who had been admitted to the Narcological Clinic “Nishati” in Tbilisi, Georgia. One hundred and three alcohol-addicted and fifty-nine opioid-addicted appropriately eligible participants (40 ± 15-year-old males) were recruited. The study was focused on the male population. Patients were split into two groups: those with an alcohol addiction and those with an opioid addiction. There were two subgroups for each group: (1) intoxication state and (2) withdrawal state. The number of matched patients in each subgroup was approximately equal. A control group was recruited from 15 healthy adults (35 ± 10-year-old males) who did not abuse drugs or alcohol and had not consumed alcohol in the previous month.

The following are exclusion criteria because they are linked to MMPs as contributing factors to the circumstances of the disease: history of a massive stroke, active malignancy within the last five years, diabetes, heart failure, liver failure, recent major surgery, and serious mental illness.

The International Classification of Diseases (ICD) criteria were used to diagnose the participants with alcohol and drug addiction. To ascertain the state of withdrawal, physicians employed anamnestic data and clinical measurements (mydriasis, rhinorrhea, diarrhea, increased lacrimation, hyperhidrosis, arthralgia, myalgia, hypertension, tachycardia, etc.). Urine was used as a biological material where the amounts of alcohol and opioids were determined. The different states of alcohol intoxication have been classified based on urine alcohol content.

The two types of opioids (methadone and buprenorphine) were detected in the urine of patients with opioid addiction in the intoxication state. The three types of opioids detected in the urine of patients going through the withdrawal stage of opioid addiction were methadone, buprenorphine, and heroin. The clinic offered anonymous, voluntary placements (treatments).

Heparinized sterile tubes (Lithium heparin, Italy) were used to collect blood samples from control group participants and from patients with alcohol and opioid addiction as soon as they were admitted to the clinic. For further plasma analyses, blood samples were centrifuged for 10 min at 3000 rpm.

### 2.2. Human Ethics

In accordance with Georgia law, signing the participation form signifies informed consent. The Chairman of the National Council on Bioethics (Tbilisi State Medical University) authorized the consent form.

All methods were carried out in accordance with Article 109e of the Georgia Law “On Health Protection”, which permits medical–biological research that is regulated under Chapter XIX of that legislation.

All experimental protocols were approved by the Ivane Javakhishvili Tbilisi State University Research and Development Service, which controls the ethical assessment of biomedical research (protocol code #8-2022/101 on 22 December 2022).

### 2.3. MMP-9 Concentration in Plasma

The Human Matrix Metalloproteinase 9 (MMP-9) ELISA kit (Catalog No. abx050165; Abbexa, UK) has been used to quantify MMP-9 concentration in plasma following the manufacturer’s instructions. This kit is based on monoclonal antibodies to human MMP-9 and contains reagents for the spectrophotometric determination of MMP-9 concentration in plasma. The MMP-9 concentration in the standards ranges from 10 to 0.16 ng/mL. SmartReader96 (Accuris™ Instruments, USA) was used to measure the color generated at 450 nm.

### 2.4. Methods of Analysis

Using Origin for Windows, version OriginPro9, the values of MMP-9 in plasma were displayed as means, medians and standard deviation (SD) and presented in the tables. MMP-9 values were compared between groups by pairwise Mann–Whitney tests (https://www.socscistatistics.com/tests/mannwhitney/default2.aspx, accessed on 2 November 2024) and shown as *p*-values in the figures. *p*-values less than 0.05 were considered statistically significant; *p*-values less than 0.01 indicated a substantial difference; and *p*-values of more than 0.05 were not considered statistically significant.

## 3. Results

### 3.1. MMP-9 in Plasma of Alcohol-Addicted Patients

The quantitative assessment of MMP-9 plasma concentration, which is achieved by the use of monoclonal antibodies against human MMP-9, encompasses all forms of MMP-9 that are present in plasma, including those which are inactive, active, and inhibited through the formation of a complex with a tissue inhibitor of metalloproteinases (TIMP).

**Figure 1:**
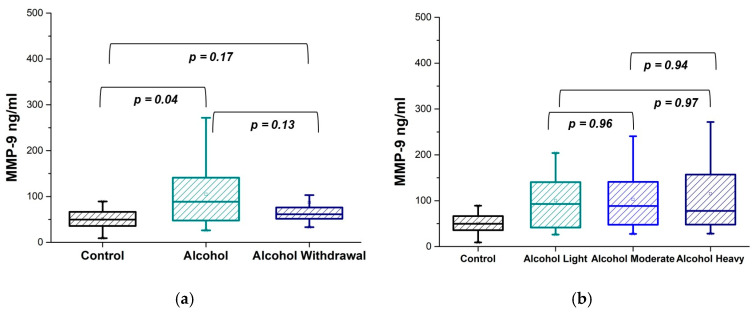
Plasma levels of MMP-9 in alcohol addicted patients and a control group of healthy volunteers. MMP-9 concentration in alcohol intoxication (Alcohol), withdrawal (Alcohol Withdrawal) states, and control group (Control) (**a**). MMP-9 concentration in three different intoxication states: light, moderate and heavy (**b**). Values are analyzed using the Mann–Whitney *U* test. *p*-values less than 0.05 were considered statistically significant; *p*-values less than 0.01 indicated a substantial difference; and *p*-values more than 0.05 were not considered statistically significant.

It is problematic to base the study on the amount of alcohol consumed, especially considering its diversity. Based on the results of a neurological examination, patients who visited the clinic were divided into two groups: patients in a state of intoxication and patients in a state of withdrawal. The comparison of MMP-9 concentrations in patients during intoxication and withdrawal states and a control group of healthy individuals is demonstrated in Figure 1a, where the *p*-values between the patients in alcohol intoxication (Alcohol) and withdrawal phases (Alcohol Withdrawal) and control individuals (Control) are presented. Table 1 represents the MMP-9 concentration in substance-abused patients expressed as mean, median, and SD.

As follows from Figure 1a and Table 1, an increase in MMP-9 concentration in plasma of alcohol-intoxicated individuals is observed in comparison with healthy individuals. Patients experiencing alcohol withdrawal are distinguished by a reduced and more evenly distributed MMP-9 concentration. The amount of MMP-9 in the alcohol withdrawal condition is approximately the same as the control, according to the data. However, statistically, no significant difference between MMP-9 concentrations in these two alcohol phases was documented.

In addition, we classified patients according to the severity of alcohol intoxication, which is based on the alcohol concentration in urine. An alcohol concentration of 0.4% is assigned as light intoxication; an alcohol concentration of 0.8% is assigned as moderate intoxication; and an alcohol concentration of 3% is assigned as heavy intoxication. Figure 1b shows that the MMP-9 concentration in the plasma of patients throughout all alcohol intoxication categories is increased in comparison with healthy individuals. The fact that MMP-9 concentration does not exhibit substantial variation is confirmed by the *p*-values for alcohol-dependent patients in several states of intoxication: light (Alcohol L), moderate (Alcohol M), and heavy (Alcohol H). Table 2 represents the MMP-9 concentration in three different alcohol intoxication states expressed as mean, median, and SD.

### 3.2. MMP-9 in Plasma of Opioid-Addicted Patients

**Figure 2:**
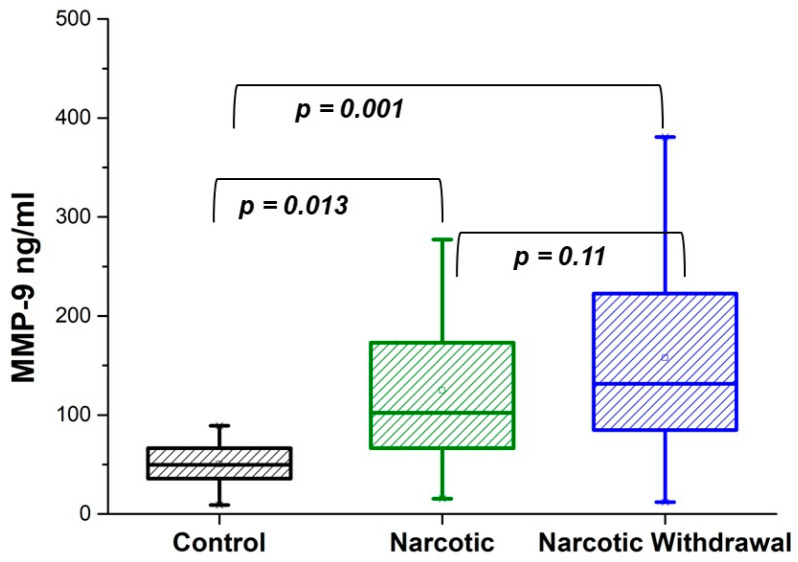
Plasma levels of MMP-9 in opioid-addicted patients and a control group of healthy volunteers. MMP-9 concentration in opioid intoxication (Narcotic), withdrawal (Narcotic Withdrawal) states, and control group (Control). Values are analyzed using the Mann–Whitney *U* test. *p*-values less than 0.05 were considered statistically significant; *p*-values less than 0.01 indicated a substantial difference; and *p*-values more than 0.05 were not considered statistically significant.

Since opioids are the most commonly available narcotic substance in our area (Georgia), the study population is represented the patients with opioid addiction. Similarly to alcohol addiction, individuals with opioid addiction were examined throughout both the intoxication and withdrawal phases. According to Figure 2 and Table 1, patients in both phases of the study had plasma concentrations of MMP-9 that were similar and increased in comparison with healthy individuals.

**Figure 3:**
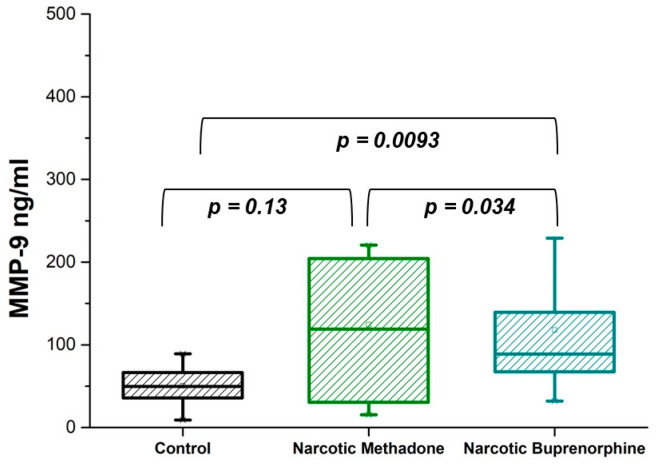
Plasma levels of MMP-9 in opioid-addicted patients and a control group of healthy volunteers. MMP-9 concentration in methadone intoxication (Narcotic Methadone), buprenorphine intoxication (Narcotic Buprenorphine) states, and control group (Control). Values are analyzed using the Mann–Whitney *U* test. *p*-values less than 0.05 were considered statistically significant; *p*-values less than 0.01 indicated a substantial difference; and *p*-values more than 0.05 were not considered statistically significant.

All patients in the intoxication state were within a medication program featuring methadone or buprenorphine. These are two often-prescribed medications that have been authorized by the US Food and Drug Administration, as they are the most effective for the treatment of opioid maintenance [34]. The results are presented in Figure 3. A significant difference was found in the MMP-9 content in the plasma of patients on methadone and buprenorphine treatment (*p* = 0.034 < 0.05). Table 3 represents the MMP-9 concentration in two different opioid intoxication states expressed as mean, median, and SD.

**Figure 4:**
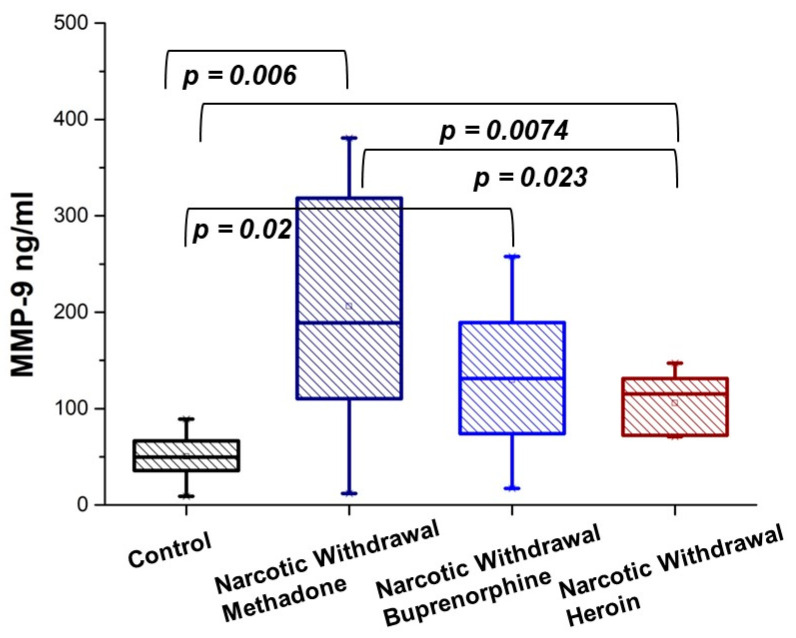
Plasma levels of MMP-9 in opioid-addicted patients and a control group of healthy volunteers. MMP-9 concentration in methadone withdrawal (Narcotic Withdrawal Methadone), buprenorphine withdrawal (Narcotic Buprenorphine Withdrawal), heroin withdrawal (Narcotic Heroin Withdrawal) states, and control group (Control). Values are analyzed using the Mann–Whitney *U* test. *p*-values less than 0.05 were considered statistically significant; *p*-values less than 0.01 indicated a substantial difference; and *p*-values more than 0.05 were not considered statistically significant.

In the cohort of patients in withdrawal, there were patients who had undergone treatment for methadone, buprenorphine and after heroin consumption. Figure 4 shows that MMP-9 levels in the case of methadone and buprenorphine are very high and that the variation in values is very large. Regarding the withdrawal after heroin consumption, the distribution of MMP-9 values is more even; the *p*-value between methadone and heroin withdrawal is *p* = 0.023 < 0.05, which indicates a significant difference. Table 3 represents the MMP-9 concentration in three different opioid withdrawal states expressed as mean, median, and SD.

### 3.3. Comparison of MMP-9 in Plasma in Alcohol and Opioid-Addicted Patients

The comparison of MMP-9 levels in the plasma of patients with alcohol and opioid dependence is shown through *p*-values in Table 4. MMP-9 concentrations in alcohol-dependent and opioid-dependent patients are comparable and significantly exceed the normal concentration. In terms of the withdrawal state, they differ greatly. MMP-9 levels stay quite high during opioid withdrawal, although they are nearly normal during alcohol withdrawal.

## 4. Discussion

MMP-9 and oxidative stress are important factors in the development of neurodegenerative disorders [35,36,37]. Oxidative stress relates to the brain shrinkage that accompanies an alteration of the range of normal brain function [37,38]. Higher MMP-9 levels have also been linked to a higher risk of cognitive impairments, according to clinical results. A rise in blood levels in regard to MMP-9 is correlated with a number of cognitive impairments linked to diseases such psychoses, schizophrenia, and epilepsy [35,36,39]. MMP-9 has been linked to conditions including dementia, post-traumatic stress disorder, bipolar disorder, and depression [40,41]. Prolonged alcohol consumption results in different neurological, cognitive, and psychological disorders [37]. For many years, dementia and depression, as well as aberrant synaptic plasticity, have been understood to be severe types of alcohol-related cognitive impairments. According to a publication by Seitz-Holland et al. from 2024, early phase psychosis is associated with a consistent rise in MMP-9 levels in plasma [36]. It is important to note that the primary limitation of the study, according to the authors, is the lack of assessment of potentially relevant factors, such alcohol or drug consumption, that can modify MMP-9 activation.

According to the obtained results, MMP-9 levels in the plasma of patients suffering from alcohol dependency are much higher than the MMP-9 concentrations of the apparently healthy donors, irrespective of the level of intoxication. Our results correlate with data from human studies, which show that MMP-9 levels are elevated in the serums of alcohol use disorder (AUD) patients [33]. When comparing MMP-9 levels in individuals experiencing alcohol intoxication and withdrawal, even more interesting results were revealed. MMP-9 in a withdrawal state is nearly identical to normal, and it is lower than the total amount of MMP-9 in an intoxication state. Alcohol shrinks brain tissue without significantly losing neurons; as a result, alcohol may be able to slow, stop, or even reverse the effects of alcohol on the brain. Disturbed neuronal function or connections can be restored during the withdrawal [42]. It is known that MMP activity may be controlled by reactive oxygen species (ROS); however, the exact mechanisms driving the regulation of MMP expression are still mostly unclear [12]. The obtained data suggest that the redox imbalance normalizes over time upon alcohol cessation, which may be the primary reason for the lack of increase in MMP-9 levels during alcohol withdrawal, as oxidative stress significantly decreases during alcohol withdrawal due to the activation of the antioxidant defense system [43].

MMP-9 has been involved in addiction to such diverse drugs as cocaine, methamphetamine, and opioids. Various studies have also implicated neuroinflammatory processes that involve MMP-9 in the pathophysiology of drug addiction [44,45]. Data from human studies indicate that its levels are increased in the hippocampus of cocaine [46] and heroin abusers [47]. Furthermore, MMP-9 mRNA levels are increased in methamphetamine addiction [48].

The selection of medications of opioid dependence is based on how effectively drugs interact with opioid receptors, as drugs, producing the associated effects [49], stimulate opioid receptors. Methadone and buprenorphine are synthetic opioids. Buprenorphine causes fewer euphoric sensations as well as less respiratory depression, making it safer than methadone but sufficient to manage opioid withdrawal [50]. The MMP-9 concentration varies across methadone and buprenorphine intoxication cases, indicating elevated MMP-9 levels in both post-exposure conditions relative to the control. The only withdrawal that exhibits lower MMP-9 levels than those seen through methadone and buprenorphine withdrawal is heroin withdrawal.

Contrary to the effects of alcohol, alterations in brain function in individuals with opioid use disorder last for an extended period, even after they discontinue using drugs [50]. The metabolism of opioids produces reactive metabolites or free radicals [51,52,53,54]. Simultaneously with reactive metabolites, ROS arise as a byproduct [54,55]. Consequently, oxidative stress and opioid dependency are linked in a feedback loop that results in long-term alterations in brain function. This enables us to propose that this cyclical relationship could sustain a high level of MMP-9 throughout withdrawal in individuals with opioid addiction, in contrast to alcohol withdrawal.

## 5. Limitations

The study concentrates on the male population due to national specificity. In Georgia, the prevalence of alcohol and opioid dependence among women is statistically significantly lower than that among men, and their frequency of consulting with doctors is considerably less.

## 6. Conclusions

This study assesses plasma MMP-9 levels as a biomarker for the phases of alcohol and opioid addiction in people with substance use disorders. In the intoxication phase, MMP-9 levels in patients with alcohol and opioid dependence are very similar and elevated compared to the control group. While the MMP-9 level nears the control level after alcohol withdrawal, it persists at a high level during three withdrawals, namely methadone, buprenorphine, and abuse of heroin.

## Figures and Tables

**Table 1:** Plasma levels of MMP-9 in substance-dependent patients and age-matched controls. Values are analyzed using Origin for Windows, version OriginPro9, and presented as means ± SD; (numbers in the parentheses are medians).

	Control	Alcohol	Alcohol Withdrawal	Narcotic	Narcotic Withdrawal
MMP-9 ng/mL	50.3 (49.7) ± 25.2	104.9 (88.7) ± 72.8	86.7 (61.3) ± 43.1	124.9 (102.1) ± 89.6	157.8 (131.6) ± 98.6

**Table 2:** Plasma levels of MMP-9 in alcohol-addicted patients in three different intoxication states: light (Alcohol L); moderate (Alcohol M) and heavy (Alcohol H). Values are analyzed using Origin for Windows, version OriginPro9, and presented as means ± SD; (numbers in the parentheses are medians).

	Alcohol L	Alcohol M	Alcohol H
MMP-9 ng/mL	100.3 (93.0) ± 59.6	102.9 (88.7) ± 67.5	115.3 (77.7) ± 99.4

**Table 3:** Plasma levels of MMP-9 in opioid-addicted patients in intoxication states: methadone (Methadone) and buprenorphine (Buprenorphine); in withdrawal states: methadone (Methadone Withdrawal), buprenorphine (Buprenorphine Withdrawal), and heroin (Heroin Withdrawal). Values are analyzed using Origin for Windows, version OriginPro9, and presented as means ± SD; (numbers in the parentheses are medians).

	Methadone	Buprenorphine	Methadone Withdrawal	Buprenorphine Withdrawal	Heroin Withdrawal
MMP-9 ng/mL	124.2 (119) ± 81.3	118 (89) ± 88	206 (189) ± 124.5	130 (131.2) ± 80	105.7 (115.4) ± 30

**Table 4:** Comparison of plasma MMP-9 concentration *p*-values between patients with alcohol and opioid addiction in intoxication and withdrawal phases.

	Alcohol and Narcotic	Alcohol Withdrawal and Narcotic Withdrawal
MMP-9 ng/mL	0.29 (are not significantly different *p* > 0.05)	0.00016 (are significantly different *p* < 0.01)

## Data Availability

The data are available from the corresponding author.
